# Perceived training load and jumping responses following nine weeks of a competitive period in young female basketball players

**DOI:** 10.7717/peerj.5225

**Published:** 2018-07-18

**Authors:** Igor de Freitas Cruz, Lucas Adriano Pereira, Ronaldo Kobal, Katia Kitamura, Cristiano Cedra, Irineu Loturco, Cesar Cavinato Cal Abad

**Affiliations:** 1 ADC BRADESCO, Osasco, SP, Brazil; 2 NAR—Nucleus of High Performance in Sport, São Paulo, SP, Brazil

**Keywords:** Training load monitoring, Team sports, Sports performance, Vertical jump

## Abstract

The aims of this study were to describe the session rating of perceived exertion (sRPE), total quality recovery (TQR), and variations in countermovement jump (CMJ) height throughout nine weeks of a competitive period in young female basketball players. In total, 10 young female basketball players (17.2 ± 0.4 years; 71.8 ± 15.0 kg; 177.2 ± 9.5 cm) participated in this study. The sRPE and TQR were assessed in each training session, whereas the CMJ height was assessed prior to the first weekly training session. The magnitude-based inferences method was used to compare the sRPE, TQR, and CMJ height across the nine weeks of training. The training loads accumulated in weeks 1, 2, and 3 were likely to almost certainly be higher than in the following weeks (ES varying from 0.67 to 2.55). The CMJ height in week 1 was very likely to be lower than in weeks 2, 5, 7, and 8 (ES varying from 0.24 to 0.34), while the CMJ height of the 9th week was likely to almost certainly be higher than all previous weeks of training (ES varying from 0.70 to 1.10). Accordingly, it was observed that when higher training loads were accumulated, both CMJ and TQR presented lower values than those presented during periods with lower internal training loads. These results highlight the importance of using a comprehensive and multivariate approach to effectively monitor the physical performance of young athletes.

## Introduction

Basketball is a high-intensity intermittent sport which requires different technical and physical abilities from the players to cope with match demands (Cormery, Marcil & Bouvard, 2008; Scanlan et al., 2012). Due to its multifaceted characteristics and extensive tournament schedule, basketball requires effective training strategies, allowing athletes to develop and maintain their optimal performance throughout the season. Due to the excessive stress and fatigue experienced by these athletes during training and matches, it is essential to adequately monitor training loads to avoid maladaptations and reduce the risk of athletic injuries (Kentta & Hassmen, 1998; Kellmann, 2010; Montgomery, Pyne & Minahan, 2010). This is especially important for young athletes who need to focus on their prospective development, aiming to achieve competitive success in their professional careers (Leite & Sampaio, 2012; Lloyd & Oliver, 2012; Balyi, Way & Higgs, 2013).

The session rating of perceived exertion (sRPE) is one of the most popular and applied methods for internal training load monitoring (Impellizzeri et al., 2004; Manzi et al., 2010; Freitas et al., 2014; Nunes et al., 2014; Nakamura et al., 2016a; Nakamura et al., 2016b). Originally suggested by Foster et al. (2001) the sRPE is a valid, reliable, and low-cost method extensively used in many different sports (Impellizzeri et al., 2004; Wallace, Slattery & Coutts, 2009; Montgomery, Pyne & Minahan, 2010; Scanlan et al., 2012; Nakamura et al., 2016b). Indeed, when assessed by means of sRPE, the internal training load seems to be related to the fitness level of professional futsal players (Milanez et al., 2011). In practical terms, players with higher aerobic fitness may present lower sRPE values than their weaker peers (Milanez et al., 2011). Furthermore, rugby union players who experienced higher cumulative training loads across a period of four weeks (>8,651 arbitrary units [AU]) were more susceptible to injury (Cross et al., 2016). That said, monitoring of training loads is important to adjust training schedules according to individual fitness levels, as well as to reduce the risk of injury.

Structured by Kentta & Hassmen (1998), the total quality of recovery (TQR) is an effective method to assess the recovery status of athletes, evaluating the relationships between “stress (experienced during training routines) and recovery”. For example, concomitant increments in creatine kinase (CK) concentrations and TQR impairment have been found in volleyball athletes (Freitas et al., 2014). Similarly, TQR has been shown to be sensitive to detect changes in stress and recovery after periods of high-intensity training (Gathercole, Sporer & Stellingwerff, 2015). In this context, constant monitoring of the balance between stress and recovery using practical and time-saving tests has been suggested in the literature, allowing more detailed comprehension of the athletes’ training status (Brink et al., 2010).

The countermovement jump (CMJ) has been used not only to measure training responses, but also to detect neuromuscular fatigue in top-level athletes (Gathercole, Sporer & Stellingwerff, 2015; Wiewelhove et al., 2015; Nakamura et al., 2016a; Kennedy & Drake, 2017; Loturco et al., 2017). For example, Delextrat, Trochym & Calleja-Gonzalez (2012) reported significant reductions in CMJ performance (from 12.6% to 19.6% in CMJ height) across a typical in-season training week in national-level female basketball players. In addition, Loturco et al. (2017) demonstrated that the meaningful variations in CMJ height (pre and post training values) analyzed throughout a competitive week can be used to adjust the training content, and thus maximize the physical performance of elite rugby union players. From these studies, it could be inferred that monitoring these indicators (i.e., internal training load and neuromechanical performance) plays a critical role in maintaining and improving athletic performance.

Surprisingly, there is a scarcity of studies investigating long-term responses in sRPE, TQR, and CMJ performance in young female basketball players (Aderson et al., 2003; Delextrat, Trochym & Calleja-Gonzalez, 2012; Nunes et al., 2014). For this population (i.e., youth athletes), monitoring training load becomes important not only to allow them to cope with training and match demands, but also to avoid possible disturbances during their prospective development (Leite & Sampaio, 2012; Lloyd & Oliver, 2012; Balyi, Way & Higgs, 2013). Thus, this study aimed to describe and examine the perceived training loads (by means of sRPE), recovery (by means of TQR), and CMJ responses across nine weeks of a competitive period in national-level young female basketball players.

## Materials and Methods

### Study design

This descriptive longitudinal study aimed to describe the TQR, sRPE, and CMJ responses across the nine weeks of a competitive season in young female basketball players. During this nine-week phase, the athletes were involved in the most important championship of the season (under-18 São Paulo State Tournament). In this period, the training schedule was planned by the technical staff to provide a good balance between stress and recovery, allowing players to cope well with the physical and physiological demands over the entire season. Typically, the training sessions started with a general warm-up (5–10 min), stretching (15 min), and *core* exercises (15–20 min). The technical-tactical training comprised game-based training drills and technical exercises (e.g., free throws, jump shot, passing drills), lasting between 60 and 90 min per session. Strength-power training encompassed plyometric (<60 contacts per session) and ballistic exercises, with light to moderate loads, using lower (i.e., jump squats) and upper-body (i.e., bench press) exercises (∼six series of six repetitions per exercise). A schematic presentation of the training schedule over the nine weeks is presented in Table 1. The TQR was assessed prior to the start of the training sessions while the sRPE was calculated after each training period across the observational period. The vertical jump height was measured once a week, prior to the first weekly training session. All athletes were previously familiarized with the testing procedures of the study due to their constant physical assessments and use of these training load monitoring tools.

**Table 1:** Schematic presentation of the training schedule across the nine weeks of training of young basketball players.

	Week 1	Week 2	Week 3	Week 4	Week 5	Week 6	Week 7	Week 8	Week 9
Matches (*n*)	–	–	1	2	2	1	2	2	1
Tec/Tac (min)	360	500	420	180	360	285	240	255	210
Strength (min)	0	120	120	60	60	120	60	60	120

**Notes.**

Tec/Tac: technical and tactical training involved game-based drills and specific technical exercises (e.g., free throws, jump shot, passing drills).

### Subjects

Ten young female basketball players (age: 17.2 ± 0.4 years; weight: 71.8 ± 15.0 kg; height: 177.2 ± 9.5 cm) from the same team, volunteered to participate in the study. The basketball team was involved in the most important youth State level competitions in Brazil and five athletes were part of the national team. The study was approved by the Anhanguera-Bandeirante University Ethics Committee (registration number 949.022) and all subjects and their legal guardians were informed of the inherent risks and benefits of the study before signing an informed consent form.

### Session rating of perceived exertion

The internal training load was recorded using the sRPE method (Foster et al., 2001). Approximately 15 min following the completion of every training session, the players were required to report the intensity of the whole session by means of a 10-point rating of perceived exertion scale proposed by Borg (1982). This value was multiplied by the respective total duration of each training session. Daily and weekly sRPE values were used for the analyses.

### Total recovery quality

The general perceived recovery was obtained in the morning before each training session using the TQR scale (Kentta & Hassmen, 1998). Basketball players were asked to report how they felt about their general recovery in relation to the last 24-h (including night sleep). The TQR scores vary between 6 and 20 with the lowest values representing poorer recovery, while the highest values represent a good recovery state.

### Countermovement jump

Vertical jumping height was determined using the CMJ. To perform this jump test, the players were instructed to execute a downward movement followed by a complete extension of the legs and were free to determine the countermovement amplitude to avoid changes in jumping coordination. All attempts were executed with the hands fixed on the hips and the players were encouraged to jump high and fast. The CMJ was performed on a contact platform (SysJump, Systware®—Brazil). A total of five attempts were allowed, interspersed with ≈15 s. The highest jump height was retained for analysis.

### Statistical analysis

The normality of the data was checked using the Shapiro–Wilk test. Due to the normal distribution, data are described as mean and standard deviation (SD). To compare the differences in the CMJ performance and accumulated training loads over the nine weeks of training, the magnitude-based inference was used (Batterham & Hopkins, 2006). The quantitative chances of finding differences in the variables tested were assessed qualitatively as follows: <1%, almost certainly not; 1%–5%, very unlikely; 5%–25%, unlikely; 25%–75%, possible; 75%–95%, likely; 95%–99%, very likely; >99%, almost certain. If the chances of having better and poorer results were both >5%, the true difference was rated as unclear. A likely difference (>75%) was considered as the minimum threshold to detect meaningful differences due to the lower probability of an error occurring in this range of probabilities to find positive/negative effects (Hopkins & Batterham, 2016). The standardized differences for the comparisons in all variables were analyzed using the Cohen’s *d* effect size (ES) (Hopkins et al., 2009).The magnitudes of the ES were qualitatively interpreted using the following thresholds: <0.2, trivial; 0.2–0.6, small; 0.6–1.2, moderate; 1.2–2.0, large; 2.0–4.0, very large and; >4.0, nearly perfect (Hopkins et al., 2009). To analyze the daily variation in the perceived training loads and TQR scores, terms such as possibly and unclear were used if the 90% confidence limits (CL) crossed one or both smallest worthwhile change (SWC; calculated by using 0.3 × coefficient of variation) boundaries (Hopkins et al., 2009), respectively. Otherwise, if the CL did not cross SWC boundaries, the effect was inferred as probable. Lastly, a Pearson product-moment coefficient of correlation was used to analyze the relationships between CMJ height, sRPE loads, and TQR scores. The thresholds used to qualitatively assess the correlations were based on the following criteria: <0.1, trivial; 0.1–0.3, small; 0.3–0.5, moderate; 0.5–0.7, large; 0.7–0.9, very large; >0.9 nearly perfect (Hopkins et al., 2009). The significance level was set as *P* < 0.05.

## Results

**Figure 1:**
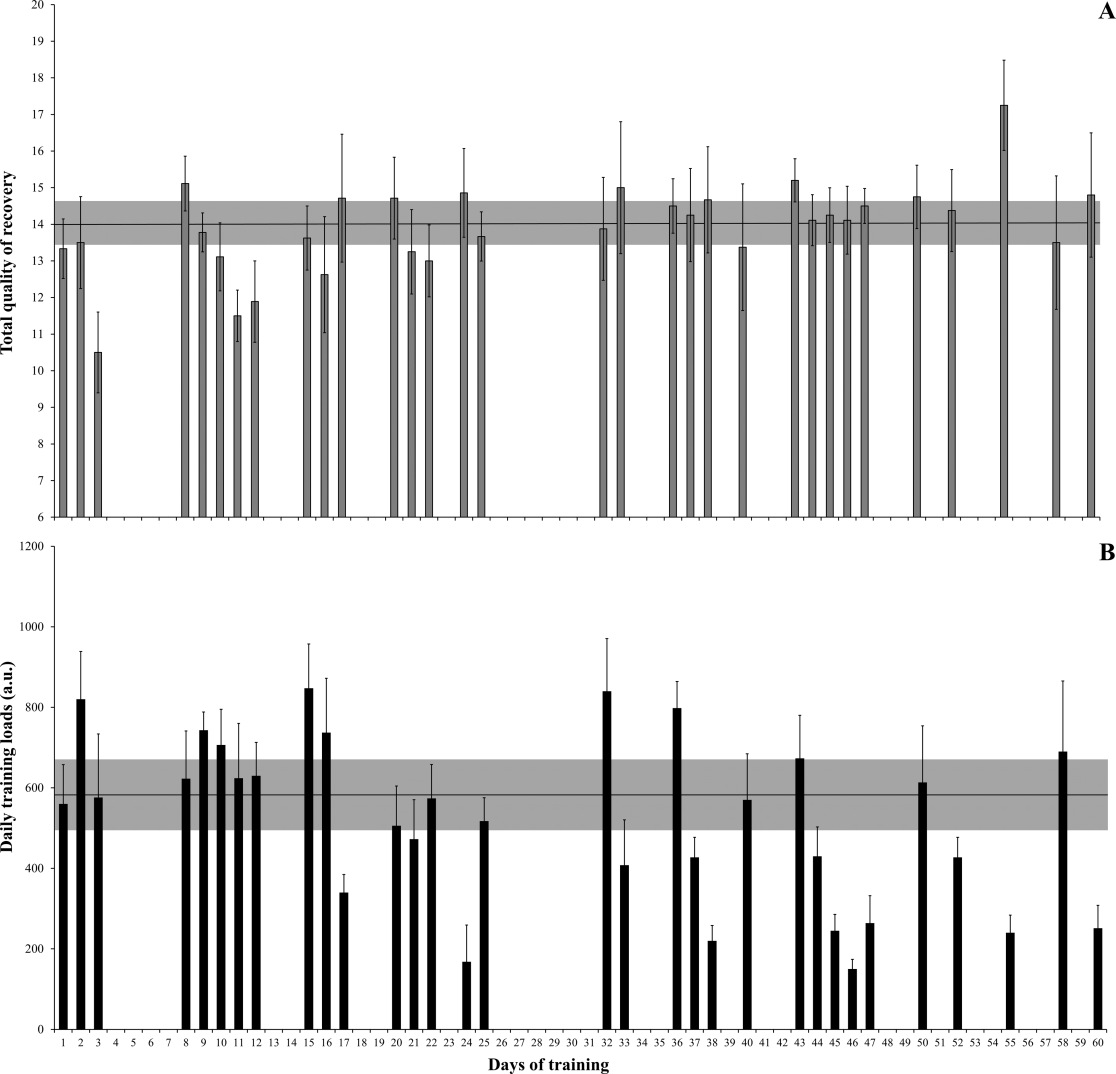
Daily training loads and the respective perceived recovery over the 60 days of training load monitoring. Total quality of perceived recovery (A) and daily training loads (B) over the 60 days of training load monitoring. In both (A) and (B) the error bars represent 90% confidence limits (CL) and the grey area represents the smallest worthwhile change (SWC), calculated by 0.3 × coefficient of variation. The terms possibly and unclear were used if the CL crossed one or both SWC boundaries, respectively. Athletes presented higher recovery scores from the 50th to 55th days (A), while higher training loads were accumulated from the 8th to 12th days (B).

The daily training loads (with their respective perceived recovery over the 60 days of load monitoring) are reported in Fig. 1. It can be observed that when higher training loads were accumulated (e.g., from the 8th to 12th days), the perceived recovery was progressively reduced. Meanwhile, in periods of lower training loads, athletes presented higher recovery scores (e.g., from the 50th to 55th days).

**Figure 2:**
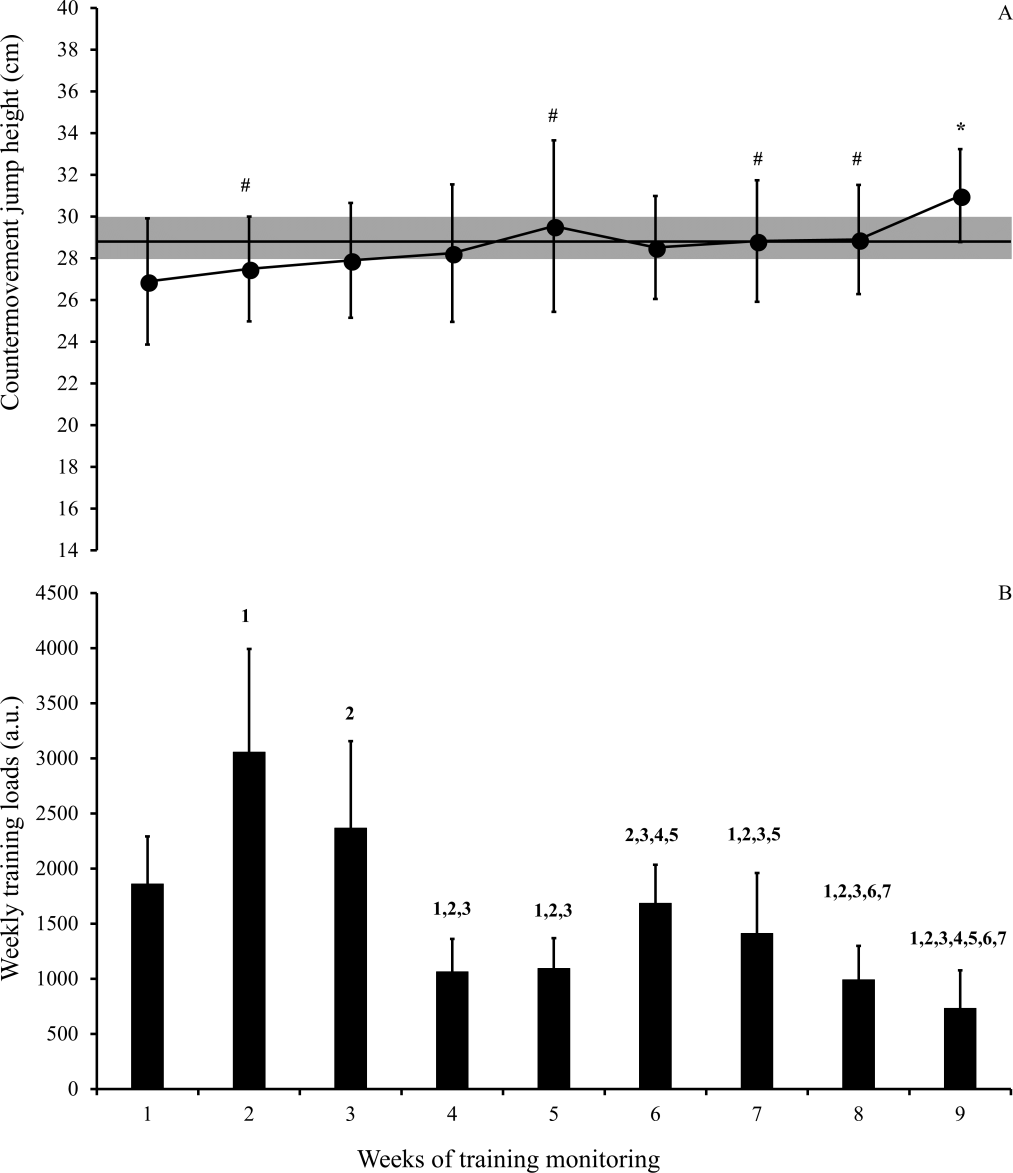
Countermovement jump height (A) and weekly internal training loads (B) across the nine weeks of the competitive period in young basketball players. (A) Error bars represent 90% confidence limits and the grey area represents the smallest worthwhile change (SWC), calculated by 0.3 × group standard deviation of the pre-values. Lower CMJ performance was found at the start of the season from weeks 1 to 3 and higher CMJ performance was observed in the final week. ^#^meaningful difference from week 1; *meaningful difference from all previous weeks. (B) The error bars represent standard deviations and the numbers represent meaningful differences from the correspondent weeks. In weeks 2 and 3, athletes accumulated the highest training loads, while the lowest training load was observed in the final (9th) week.

Figure 2 depicts CMJ performance (Fig. 2A) and perceived training loads (Fig. 2B) across the nine weeks of the competitive period in young basketball players. The CMJ height in week 1 was likely to very likely lower than weeks 2, 5, 7, and 8 (ES varying from 0.24 to 0.34), while the CMJ performance was likely to almost certainly higher in the 9th week than all previous weeks of training (ES varying from 0.70 to 1.10) (Fig. 2A). The following comparisons between the weeks for CMJ performance were all rated as unclear. In relation to the comparison of the weekly training loads (Fig. 2B), the sRPE accumulated in weeks 1, 2, and 3 were likely to almost certainly higher than the following weeks (ES varying from 0.67 to 2.55), except for the comparison between weeks 1 and 6 when a meaningful difference was not observed. Small and significant correlations were observed between sRPE and CMJ height (*r* =  − 0.28; *P* < 0.05) and TQR scores (*r* =  − 0.25; *P* < 0.05). In addition, a moderate correlation was observed between the CMJ height and TQR scores (*r* = 0.37; *P* < 0.05).

## Discussion

The aims of this study were to describe the internal training load, perceived recovery, and variations in jumping performance throughout nine weeks of competition in national level young female basketball players. After analyzing the data, we observed that when higher training loads were accumulated (e.g., week 2), both CMJ and TQR presented lower values than those presented during periods with lower internal training loads (e.g., week 9, where higher jump performances and lower TQR scores were identified). These results highlight the importance of using a comprehensive and multivariate approach to effectively monitor the physical performance of young athletes.

The internal training loads accumulated over the nine weeks of competitive period in this study (mean weekly internal training load: 1584.3 ± 237.4 a.u.) are close to those observed in U16 and amateur basketball players (Otaegi & Los Arcos, in press; Ferioli et al., 2018b), but lower than those reported in elite senior players (Nunes et al., 2014; Ferioli et al., 2018b). However, not only the absolute training loads accumulated during a given period, but also the load distribution across the weeks and their continuous “adjustments” in accordance with player responses seem to have a crucial impact on both perceived stress and physical adaptations to training (Nunes et al., 2014; Saw, Main & Gastin, 2016; Aoki et al., 2017; Gabbett et al., 2017). Indeed, a recent systematic review demonstrated that subjective perceptions of well-being are impaired in response to acute and chronic increases in training loads (Saw, Main & Gastin, 2016). On the other hand, enhanced (and optimized) perceptions have been observed when athletes experience reduced training loads (Saw, Main & Gastin, 2016). The same holds true for national team players, where a reduced balance between recovery and stress (i.e., lower recovery/higher stress) was observed in a period of high-intensity training, and an opposite pattern was detected during the taper period (i.e., when lower training loads were experienced) (Nunes et al., 2014). A similar pattern could be observed after analyzing our data: in the first three weeks, the basketball players experienced higher training loads than the following weeks, which was accompanied by a progressive reduction in the TQR across the week. Conversely, when training loads were reduced in the final weeks, TQR values varied less, therefore presenting higher scores than those presented during the early training weeks (Fig. 1). These findings highlight the importance of promoting a good balance between recovery and stress, thus allowing athletes to adequately cope with specific training loads, reducing the risk of non-functional overreaching and injuries, and finally providing sufficient stimulus for a progressive improvement in physical performance (Halson, 2014; Nunes et al., 2014; Cross et al., 2016; Aoki et al., 2017; Gabbett et al., 2017).

In relation to the power performance, the assessment of CMJ height in basketball players seems to be a key factor, since vertical jumps are amongst the most prevalent actions performed throughout matches and technical training sessions (Ziv & Lidor, 2010). Moreover, jump performance was capable of discriminating young athletes from distinct competitive levels (Torres-Unda et al., 2013). Therefore, monitoring the variations in this important physical capacity, which is easy to implement during training routines, may provide important information for practitioners in relation to the physical performance of young basketball players. As such, the training loads may be adjusted in accordance with the variations in jump performance across a given period of training. Notably, although changes were observed in the CMJ height and TQR scores when higher training loads were accumulated, the sRPE was only weakly correlated to these variables (*r* =  − 0.28 and −0.25, respectively). This suggests that internal training loads, perceived recovery and the vertical jump height should be implemented as “complementary tools” to adequately evaluate and control athletes’ training responses throughout a given training process.

It is important to note that CMJ performance progressively increased over the nine-week period of observation, with the exception of week 6 where a variation in CMJ height could be observed (Fig. 1A). This is possibly related to the increase in the number of matches per week (from one to two matches per week), which occurred between weeks 4 and 5. Consequently, the players experienced a slight (but not meaningful) reduction in the vertical jump performance. On the other hand, after week 6, the intensity of both weekly and daily training loads was progressively reduced over the following weeks, which could induce a later improvement in CMJ performance (which occurred over the final week). Variation in jump performance in response to matches and training has been demonstrated in previous studies with different sport disciplines (Gathercole, Sporer & Stellingwerff, 2015; Malone et al., 2018; Sams et al., in press). For instance, Gathercole, Sporer & Stellingwerff (2015) reported a meaningful reduction in CMJ flight time and well-being perception after an intensified training period in elite female rugby players. In another study with soccer players, Ferioli et al. (2018a) found moderate to large negative correlations between sRPE training loads and changes in CMJ peak power after a specialized training period in basketball players. These studies emphasize the importance of frequently monitoring the neuromuscular responses to training, mainly in team-sports athletes, who commonly deal with concurrent training effects (Fyfe, Bishop & Stepto, 2014; Loturco et al., 2015). Finally, our results also suggest that the training loads were adequately programmed (and adjusted) across the nine-week competitive period, which can be confirmed by analyzing the progressive improvements in physical performance (i.e., CMJ height), which is of fundamental importance for athletes in the final stages of development (Leite & Sampaio, 2012; Lloyd & Oliver, 2012).

The main limitation of the study was that the nutritional and sleep habits of the athletes were not controlled. This is an important issue since poor sleep quality and dehydration status can negatively interfere in the physical performance of the athletes (Díaz-Castro et al., 2018; Heishman et al., 2017). Another important limitation was the small sample size and the absence of a control group. However, this study was performed with top-level young athletes during an actual competitive period, which denotes good ecological validity, providing practical and valuable information for coaches and researchers.

In summary, this study reported the typical training loads, perceived recovery, and vertical jump variations experienced by young female basketball players throughout nine weeks of a competitive period. It was detected that the perceived recovery decreased in periods where higher training loads were accumulated in relation to the previous weeks, which presented lower training loads and higher TQR scores. Furthermore, a progressive and gradual improvement in jump performance was observed across the nine weeks of training, with highest CMJ heights being observed in the 9th week.

## Conclusions and Practical Applications

The presented data provide valuable information for strength and conditioning coaches searching for practical and applied tools for effective training load monitoring. The sensitivity of such methods allows frequent and adequate adjustments in the training content, based on the internal training load accumulated and on the variations in the athletes’ perceived recovery. Accordingly, maintenance of the balance between recovery and stress (imposed by training loads) may be of fundamental importance to allow meaningful improvements in physical performance. Finally, the description of the typical internal training loads experienced by high-level young players across competitive periods can help coaches and sport scientists to appropriately define and adjust their training programs. Further studies should be conducted to examine the differences between training programs which use (or not) monitoring training tools (e.g., TQR and RPE) on a daily basis.

##  Supplemental Information

10.7717/peerj.5225/supp-1 Data S1 Raw data

## Additional Information and Declarations

### Data Availability

The following information was supplied regarding data availability: The raw data have been provided as Data S1.
